# Exploring the molecular mechanism of Gan Shuang granules for the treatment of non-alcoholic steatohepatitis using network pharmacology, molecular docking, and experimental verification

**DOI:** 10.3389/fphar.2023.1082451

**Published:** 2023-01-24

**Authors:** Guoguo Zhi, Bingjie Shao, Tianyan Zheng, Jie Mu, Jingwei Li, Yiyuan Feng, Sha Zhu, Yanni Dang, Feng Liu, Dong Wang

**Affiliations:** ^1^ School of Basic Medicine, Chengdu University of Traditional Chinese Medicine, Chengdu, Sichuan, China; ^2^ Shanxi Buchang Pharmaceutical Company Limited, Xi’an, Shanxi, China

**Keywords:** GSG, NAFLD/NASH, UHPLC-Q/Orbitrap-MS/MS, network pharmacology, molecular docking, molecular mechanism

## Abstract

**Background:** With the gradual increase in prevalence in recent years, non-alcoholic steatohepatitis (NASH) has become one of the significant health problems that urgently needs to be addressed worldwide. GanShuang Granules (GSG) is derived from the classical Chinese formula Xiaoyao San and mainly used in the clinical treatment of chronic liver diseases.

**Objective:** In this study, we aim to gain a deeper insight into the inhibiting effects of GSG on non-alcoholic fatty liver disease (NAFLD) rats and preliminarily elucidate the underlying intervention mechanisms.

**Methods:** First, High performance liquid chromatography (UHPLC-Q/Orbitrap-MS/MS) was used for the active compounds prediction in GSG. Then the data was mapped to mzCloud database. The targets corresponding to GSG compounds were collected from public databases, along with disease genes for NAFLD. The core targets and molecular mechanisms of GSG for NAFLD treatment were predicted by protein-protein interaction (PPI) network, Gene Ontology (GO), and Kyoto Encyclopedia of Genes and Genomes (KEGG) functional enrichment analyses. Molecular docking of the core target-component interactions was simulated using AutoDock Vina software. The effect of GSG on NASH rats was evaluated by pathological staining and analysis of various index results. Finally, the candidate targets were further validated by ELISA and western blot (WB) analyses.

**Results:** Combining UHPLC-Q/Orbitrap-MS/MS data analysis and public database data, a total of 346 cross-targets were obtained, corresponding to 81 compounds. The subnetwork with an MCODE score of 53.623 is a potential core target group for this study. GO and KEGG enrichment analyses showed that the targets of GSG in NAFLD were mostly related to oxidative stress, the NF-κB signaling pathway, and the apoptosis signaling pathway. By integrating the results of network pharmacology analysis, the core objectives of this study mainly include *AKT1, CASP9, TNF,* and *CASP8*. The core ingredients are related to resveratrol and fisetin. The molecular docking results indicated key binding activity between AKT1-fisetin, AKT1-Resveratrol, and CASP8-fisetin. Moreover, GSG could improve the inflammatory status and restore the abnormal lipid accumulation of NAFLD/NASH liver, and these levels are further verified by pathological staining and detection of related indicators. Mechanistically, GSG could regulate protein expression levels in the liver for P65, p-P65, IKB, p-IKB, IKK, caspase-3, -8, -9, and cytochrome C, etc. It reflects the inhibitory effect of GSG on the NF-κB/IκB signaling pathway.

**Conclusion:** Our results suggested that GSG demonstrated therapeutic effects on NAFLD/NASH rats, and these may be mainly reflected in the inhibitory effects on the NF-κB/IκB signaling pathway and its downstream inflammation and apoptosis signals.

## 1 Introduction

Non-alcoholic fatty liver disease (NAFLD) is recognized as the most common liver disease worldwide (Eslam et al., 2018). NAFLD encompasses a spectrum of liver conditions that are not caused by other etiology, such as more aggressive non-alcoholic steatohepatitis (NASH) that involves liver inflammation, fibrosis, and even to the lethal cirrhosis that may result in hepatocellular carcinoma and liver failure (Goldberg et al., 2017). Epidemiological studies have shown that NASH affects 3%–5% of the global population as of 2019 (Povsic et al., 2019). It also significantly increases the risk of cardiovascular disease (Wong and Lim, 2018). With the prevalence of NASH increasing year by year, the global public health security pressure is getting heavier (Kanwal et al., 2021). NASH has become one of the major global health issues to be addressed (Raza et al., 2021). There are no currently approved effective drugs for NASH/NAFLD. Traditional Chinese medicine (TCM) has a long history of thousands of years and has its own advantages and characteristics in personalized treatment and early intervention. Therefore, TCM has the characteristics of multi-component, multi-target, and multi-channel, which may become an indispensable component in the research and development of new drugs for multifactorial such as NAFLD/NASH (Castillo and Lieberman, 2015).

Ganshuang granules (GSG) are based on the traditional Chinese medicine formula Xiaoyao San, combined with modern pharmaceutical research results and clinical trials for liver disease. The formula contains 13 herbs, such as *Bupleuri Radix*, *Angelicae Sinensis Radix*, *Poria,* and *Salviae Miltiorrhizae Radix et Rhizoma*, which have good therapeutic effects on clinical acute and chronic hepatitis and liver cirrhosis (Shi et al., 2017; Zeng et al., 2020; Lu et al., 2021). Modern pharmacological studies indicated that GSG can regulate the intestinal microbiota and moderate CCl4-induced liver fibrosis (Zhao et al., 2021). In addition, GSG can slow the progression of liver fibrosis by impeding mTOR-autophagy to inhibit the activation of hepatic stellate cells (HSCs) (Shi et al., 2017). Despite the abundant experimental and clinical data on GSG for the treatment of NAFLD/NASH, its main active ingredients, core targets, and potential pharmacological mechanisms have not been fully elucidated.

Cyberpharmacology is an emerging interdisciplinary field (Liu et al., 2021) that is usually used to reveal drug-target-disease associations and has a wide range of applications in explaining the pharmacological mechanisms of TCM (Li and Zhang, 2013; An et al., 2020). Molecular docking is a structure-based method that helps the analysis of the interaction between the protein receptors and small molecular ligands (Li and Zhang, 2013; Liang et al., 2014). Therefore, network pharmacology and molecular docking are often used complementarily in modern pharmacological studies of herbal medicines, and their predictive results are then verified experimentally (Villoutreix et al., 2008; Xue et al., 2015). However, in operation, network pharmacology focuses on the construction of drug-disease networks with the aid of relevant databases and the analysis and screening of key compounds and targets (Liang et al., 2014). However, the disadvantages of this method are slow database updates and the fact that the actual composition of the herbal complex is not equal to the sum of the herbal ingredients in the composition database (Ru et al., 2014). At present, UHPLC-Q/Orbitrap-MS/MS is mainly applied to the multicomponent detection of complex samples and is widely used in the determination of TCM compounds and in other fields (Gao et al., 2020). Therefore, the combination of UHPLC-Q/ORBITRAP-MS/MS and network pharmacology can overcome the above drawbacks and improve the credibility of the research conclusion.

This study aims to use bioinformatics and experimental validation to determine the potential mechanism of GSG in the treatment of NASH and provide a basis for the clinical application of GSG. At the same time, it can also help screen the effective active compounds of GSG, improve the formulation of GSG, and provide a reliable reference for exploring the pharmacological mechanism of other Chinese medicines in the treatment of NASH.

## 2 Materials and methods

### 2.1 UHPLC-Q/Orbitrap-MS/MS analysis

#### 2.1.1 Drug solution preparation

Three grams of GSG were dissolved in 20 mL of ultrapure water and sonicated for 30 min. The mixture was allowed to cool down to room temperature, fixed, and shaken well. Then the supernatant was then centrifuged at 4°C for 10 min at 20,000 rpm, and filtered through a 0.22-µm microporous membrane. One milliliter was quantified precisely and used for subsequent composition determination.

#### 2.1.2 Chromatographic conditions

The chromatographic column was a Waters Acquity HSS T3 column with a size of 10 mm × 2.1 mm and a particle size of 1.8 µm. The aqueous and organic phases were 0.1% formic acid aqueous solution A) and 0.1% formic acid acetonitrile B), respectively. The chromatographic gradients were as follows: 95%–75% solution A, 0 min–15 min; 75%–50% solution A, 15 min–20 min; 50%–5% solution A, 20–23 min; 5% solution A, 23 min–25 min; 95% solution A, 25 min–28 min. The flow rate and the injection volume were 0.3 mL/min and 10 µL with column temperature at 40°C.

### 2.2 Network pharmacology

#### 2.2.1 Screening and target prediction of GSG active ingredients

We identified the active ingredient contained in GSG using UHPLC-Q/Orbitrap-MS/MS (Gao et al., 2020). The total ion flow chromatography of the fingerprint positive and negative ion patterns was compared with the mzCloud and mzVault databases to extract the composition information on the mzCloud best match score > 60. Subsequently, compounds with a mzCloud match score of 80 or higher were then applied for further screening (Chen et al., 2022), and gene names were calibrated by using the UniProt database (UniProt, 2019; UniProt, 2021). The Traditional Chinese Medicine Systems Pharmacology Database and Analysis Platform (TCMSP) database was used as the main data source for the analysis of component targets (Ru et al., 2014). The Bioinformatics Analysis Tool for Molecular Mechanisms of Traditional Chinese Medicine (BATMAN-TCM) and SwissTargetPrediction databases were used as supplementary data sources to complete the collection of ingredient targets without in TCSMP. The InChI and canonical SMILES of the ingredients were obtained from the PubChem database and entered into BATMAN-TCM (Liu et al., 2016) and SwissTargetPrediction databases (Daina et al., 2019) to obtain the target information corresponding to the compounds. Target screening criteria were as follows: BATMAN-TCM, score not less than 20; SwissTargetPrediction, target probability greater than 0.

#### 2.2.2 Collection of gene targets for NAFLD

The NAFLD-related human gene targets were mainly retrieved from three databases: Human genetic database (GeneCards) (He et al., 2020; McNeill, 2021), Human Online Mendelian Genetic Database (OMIM) (Amberger et al., 2015), and Genetic Pharmacology and Pharmacogenomics Database (PharmGKB) (Barbarino et al., 2018; Mitra-Ghosh et al., 2020). The gene names were calibrated using the UniProt database (UniProt, 2019; UniProt, 2021). Finally, we merged data and remove duplicate content.

#### 2.2.3 Collection of potential targets for GSG treatment of NAFLD

Venny 2.1.0 software (Wang et al., 2021) was used to draw a Venn diagram of the interaction between the component targets of GSG and the disease targets of NAFLD. The common targets of the two were used as potential targets for GSG treatment of NAFLD.

#### 2.2.4 Component-target network construction and analysis

Cytoscape 3.9.1 software (Li et al., 2022) was used for the analysis and image visualization of component-target network graphs.

#### 2.2.5 Protein-protein interaction (PPI) network construction and core gene screening

The common gene targets of GSG and NAFLD were uploaded to the STRING database (Szklarczyk et al., 2019), where the biological species were selected as “homosapiens”, the minimum interaction threshold was selected as “highest confidence” (> 0.90), and the disconnected nodes were hidden in the network. The results of the network analysis were imported into Cytoscape 3.9.1. And the MCODE plug-in was used to identify core targets (Deng et al., 2019). The images of subnetworks with MCODE scores > 5 were exported, and all targets with the highest score were obtained for further analysis.

#### 2.2.6 GO enrichment analysis and KEGG pathway analysis

The Cluster Profiler software package (Dalmaijer et al., 2022) in R4.2.1 (Thompson et al., 2012) software was used to perform GO and KEGG pathway enrichment analysis (FDR<0.05). The top 10 GO terms of biological process and the top 20 KEGG pathways were then selected to draw a column chart. Among them, the results of GO analysis mainly included cellular component (CC), molecular function (MF), and biological process (BP). Based on the related literature studies, the pathway information about NAFLD/NASH molecular mechanisms in KEGG was screened, and the top 20 pieces of information were taken and plotted in a bar graph. We integrated the data, drew a pathway-target, target-component network diagram through Cytoscape 3.9.1 software, and screened the potential core targets and compounds of GSG for NAFLD/NASH according to the degree value.

### 2.3 Molecular docking

SDF files of small molecule compounds were downloaded from the PubChem database and converted to mol2 format using Chem3D software (Liu et al., 2021). Core target data were obtained from the Protein Data Bank (PDB) (http://www.rcsb.org/), and the large molecule targets were treated with PyMol to remove the original ligands and hydrogenated with water. Finally, the data were imported into AutoDock Vina for molecular docking (Trott and Olson, 2010). Heatmaps were plotted using R software, with the horizontal coordinates representing the macromolecular proteins and the vertical coordinates representing the small molecule compounds, both less than −5 kJ mol^−1^ (Cui et al., 2022). PyMol was used to plot the 3D stereograms of target and component docking to visualize the interaction between compounds and targets (Seeliger and de Groot, 2010). The high-quality 3D structures of small molecules and proteins were exhibited by LIGPLOT + (version v2.2) software. This helped us to clearly observe the type of bonding between the ligands and protein (Shahid et al., 2021).

### 2.4 *In Vivo* experiments

#### 2.4.1 Reagents

The serum biochemical reagents included ALT (LOT: 140121005), AST (LOT: 140221004), ALP (LOT: 140321002), TC (LOT: 141621013), TG (LOT: 141721003), LDL (142021004), and HDL (LOT. 142121006). TNF-a (E-EL-R2856-96T), IL-6 (E-EL-R0015-96T), and IL-1β (E-EL-R0012-96T) ELISA kits were purchased from Elabscience Biotechnology Co., Ltd. Anti-Caspase-8 antibody (Abcam, ab108333), anti-Caspase-3 antibody (Abcam, ab184787), anti-Cytochrome C antibody (Abcam, ab133504), and anti-IKK-β antibody (Abcam, ab124957) were purchased from Abcam. BCL2 monoclonal antibody (60178-1-1 g) and GAPDH (60004-1-Ig) antibody were obtained from Proteintech Group, Inc. NF-κB p65 antibody, phospho-NF-κB p65 antibody (LOT:17), a-SMA (CST, #19245), IκB-α antibody (CST, #4812), and phospho-IκB-α antibody (CST, #2859) were provided by Cell Signaling Technology, Inc. Chemiluminescence ECL Detection Kit (P90719) was obtained from Bobst Biotechnology.

#### 2.4.2 NASH rat model

Forty 6-8-week-old male SD rats weighing 180 g ± 20 g were purchased from Beijing HFK Bioscience Co., Ltd. (Beijing, China; certification no. SCXK-Beijing-2019–0008). All rats were allowed to acclimatize for 1 week prior to the experiments and were maintained at a constant temperature (25°C ± 1°C) and 55% ± 5% humidity with a 12-h/12-h light/dark cycle. The control group was given ordinary feed (TP3622647C) purchased from Chengdu Dashuo Laboratory Animal Co., Ltd. The model group gave a choline-deficient high fat diet (CDHFD) (45% kcal from fat) (TP3622657), purchased from Trophic Animal Feed High-Tech Co., Ltd.

After one week of adaptive feeding, the rats were randomly divided into three groups: the control group (*n* = 10), the model group (*n* = 18), and the drug administration group (*n* = 12). All rats, except the control group, were fed CDHFD. At the end of 4 and 8 weeks of the experiment, three model rats and two control rats were randomly selected, fasted overnight, anesthetized with 3% pentobarbital (1 mL/kg) intraperitoneal injection, and their blood, spleen, and liver samples were collected. The model rats were assessed for pathological changes in the liver by virtue of HE and oil red O section staining. The success of the NASH model was based on the appearance of extensive steatosis and massive accumulation of inflammatory cells in the liver. After 8 weeks, the model was successful. The dose of GSG of 0.81 g/kg was calculated with reference to the “Ratio Table of Equivalent Dose of Humans and Animals Converted to Body Surface Area”. GSG treatment by gavage was given at a fixed time of 9:00 a.m. once a day for 8 weeks. The animal study was reviewed and approved by Ethics Committee of Chengdu University of Traditional Chinese Medicine. Animal studies were conducted in compliance with international regulations for the use and care of laboratory animals (Kong and Qin, 2009).

#### 2.4.3 Liver and spleen index calculation

The liver and spleen indices were calculated using the formula: liver index = liver weight/body weight and spleen index = spleen weight/body weight.

#### 2.4.4 Blood and tissue samples

Before euthanizing, the animals fasted overnight. Then, 3% pentobarbital (1 mL/kg) was injected intraperitoneally for anesthesia, blood was collected from the abdominal aorta and centrifuged at 3,000 rpm, 4°C for 15 min, and the supernatant was removed. Livers were quickly removed and stored in 4% formaldehyde for 24 h and then kept in phosphate-buffered saline at 4°C until histological processing. Four tissue blocks from different random liver locations were placed in lyophilization tubes, rapidly frozen in liquid nitrogen, and stored at −80°C until use.

#### 2.4.5 Histology

Formaldehyde-fixed tissue was paraffin-embedded, sectioned (3 µm), and subsequently stained with HE to assess hepatic steatosis, inflammation, and hepatocyte ballooning and MASSON staining to assess fibrosis. Steatosis was further assessed by sectioning (4 µm) from frozen tissue and staining with oil red O.

#### 2.4.6 Biochemistry

The levels of ALT, AST, ALP, TC, TG, LDL, and HDL in the serum were measured using a fully automated biochemical analyzer (BK-200) according to the reagent instructions.

#### 2.4.7 Enzyme-linked immunosorbent assay (ELISA)

The appropriate amount of tissue was weighed, ground with 9 times homogenization medium, centrifuged at 4°C and 3,000–4,000 rpm for 10 min, and the supernatant was taken to prepare 10% tissue homogenate. The levels of TC, TG, FFA, IL-6, IL-1β, and TNF-α in the liver homogenates were determined according to the ELISA kit instructions.

#### 2.4.8 Western blot (WB)

An appropriate amount of liver tissue was homogenized in pre-frozen lysis buffer and then left on ice for 30 min. After centrifugation at 12,000 rpm, 4°C for 15 min, the supernatant was collected as a protein sample. The proteins in the sample were separated by 10% SDS‒PAGE and transferred to a 0.45-µm PVDF membrane. The membrane was blocked with 5% skim milk for 2 h, and the primary antibody was incubated overnight at 4°C. Then, 15 min of TBST washing was performed 3 times, and the secondary antibody was incubated for 1 h at room temperature. Specific protein-antibody complexes were measured by ECL (enhanced chemiluminescence solution), and the band intensity was quantified with ImageJ. All experiments were performed in triplicate.

#### 2.4.9 Immunohistochemistry (IHC)

The liver tissue embedded in paraffin was fixed and sectioned (4 µm). The slices were placed in a 60°C oven for 120 min and then dewaxed in xylene and ethanol. Next, 3% H_2_O_2_ was incubated for 10 min at room temperature to quench endogenous peroxidase activity. Normal goat serum was used to block for 20 min. The primary antibody was incubated overnight at 4°C. The sections were then incubated with HRP-labeled goat anti-mouse and HRP-labeled goat anti-rabbit antibodies for 30 min at 37°C and washed with PBS. Immunoreactivity was observed using DAB at room temperature and restained with hematoxylin. Finally, the sections were dehydrated and sealed. The staining was visualized using a Leica digital section scanner, and the images were captured at a certain magnification by importing the 3D viewing software SlideViewer.

### 2.5 Statistical analysis

All data variables were tested for conformity to a normal distribution. Normally distributed variables are expressed as the mean ± SD. All statistical comparisons were analyzed by one-way ANOVA. *p* < 0.05 and < 0.01 were considered statistically significant.

## 3 Results

### 3.1 Chemical composition of GSG

To initially identify the chemical composition of the GSG and investigate its molecular mechanism for the treatment of NASH, this study was conducted to identify the composition of GSG by UHPLC-Q/Orbitrap-MS/MS. The total ion current chromatograms (TICCs) in negative and positive ionization modes are shown (Supplementary Figure S1). A total of 111 ingredients were matched in mzCloud with best match score > 80. The list of the top 10 compounds with mzCloud scores was nicotinic acid, resveratrol, senkyunolide H, adenosine, cis-resveratrol, DL-stachydrine, methyl caffeate, chlorogenic acid, trigonelline, and guanine (Supplementary Table S1). Interestingly, the most of the above ingredients have pharmacological effects on improving hepatic lipid accumulation and anti-inflammation. Among them, nicotinic acid and resveratrol (Che et al., 2020) were able to significantly improve the pathological changes in NAFLD and reduce lipid accumulation in the liver (Kashyap et al., 2019; Tejada et al., 2021). Methyl caffeate (Shin et al., 2004; Jantas et al., 2020; Lee et al., 2021), chlorogenic acid (Shi et al., 2013; Shi et al., 2016; He et al., 2021; Shi et al., 2021), and trigonelline (Zhang D. F. et al., 2015a; Afifi et al., 2017; Costa et al., 2020) have good biological properties of anti-inflammation, antioxidation, increased insulin secretion, protected liver function, and improved liver injury.

### 3.2 Collection of component targets for GSG and disease targets for NAFLD

A total of 111 chemical compounds were identified by UHPLC-Q/Orbitrap-MS/MS and matched using the TCMSP, Batman-TCM, and SwissTargetPrediction databases. Of these, a total of 26 ingredients do not exist in the target database. The duplicate content was removed, and a total of 85 compounds were collected, corresponding to 1,423 targets. NAFLD has 1,422 disease targets. The intersection targets of ingredients and diseases were plotted using Venny 2.1.0 software, for a total of 346 (Supplementary Figure S2). The intersecting targets were used as potential targets for GSG treatment of NAFLD in the next step of the analysis.

### 3.3 Construction of the GSG component-intersection target network map

**FIGURE 1:**
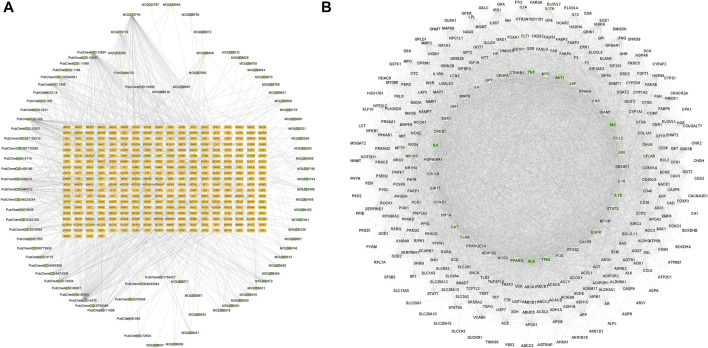
Component-target network diagram and protein-protein interaction (PPI) network. **(A)** Network diagram of GanShuang Granules (GSG) compounds corresponding to targets (orange rectangles represent targets; green dots represent compounds). **(B)** PPI diagram of the protein interactions between GSG and non-alcoholic fatty liver disease (NAFLD) intersection targets (network nodes represent proteins, line segments represent protein interrelationships; depending on the degree value, the size of the nodes changes from small to large, and the color changes from light to dark).

The 346 intersection targets and the corresponding 81 compounds were integrated and imported into Cytoscape 3.9.1 software for image visualization and analysis to construct a component-target network graph. The network graph contained a total of 434 nodes and 963 edges (Figure 1A). The 10 ingredients with the highest degree value include DL-norleucine, 4-hydroxymandelic acid, resveratrol, azelaic acid, and trans aconitic acid (Supplementary Table S2).

### 3.4 Construction of the PPI network and cluster analysis

**FIGURE 2:**
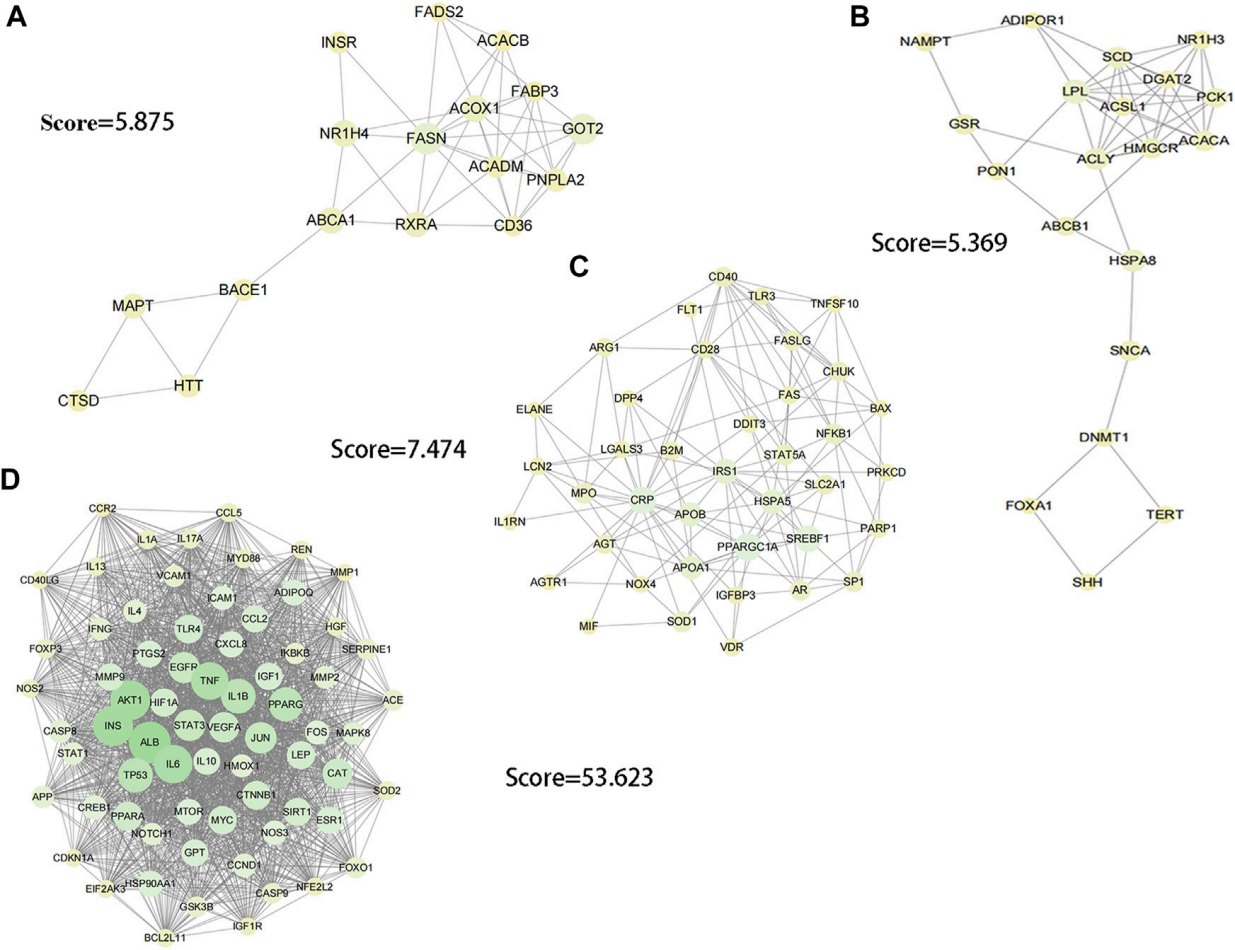
Image visualization of subnetworks with scores > 5 using the MCODE plug-in to find subnetworks. **(A)** score = 5.875. **(B)** score = 5.369. **(C)** score = 7.474. **(D)** score = 53.623.

In order to explore the core targets of GSG in the treatment of NAFLD/NASH, the intersection targets were imported into the STRING database to generate protein interactions and were imported into Cytoscape 3.9.1 software for image visualization and analysis. As shown in the figure (Figure 1B), the network has 340 nodes and 6,995 edges. The “edges” indicate the interrelationship between the intersection targets and show the degree of association. Therefore, the larger the node is, the higher the degree of association between the target and the rest of the proteins. The MCODE plug-in was used to find the subnetworks, and all the genes inside the subnetwork with the highest score were treated as hub genes (Supplementary Table S3). Four subnetworks with scores above five were 5.369, 5.875, 7.474, and 53.623, respectively (Figures 2A–D). Seventy targets contained in the subnetwork with the highest score (Figure 2D) were used as potential core targets for the next analysis.

### 3.5 GO and KEGG enrichment analysis

**FIGURE 3:**
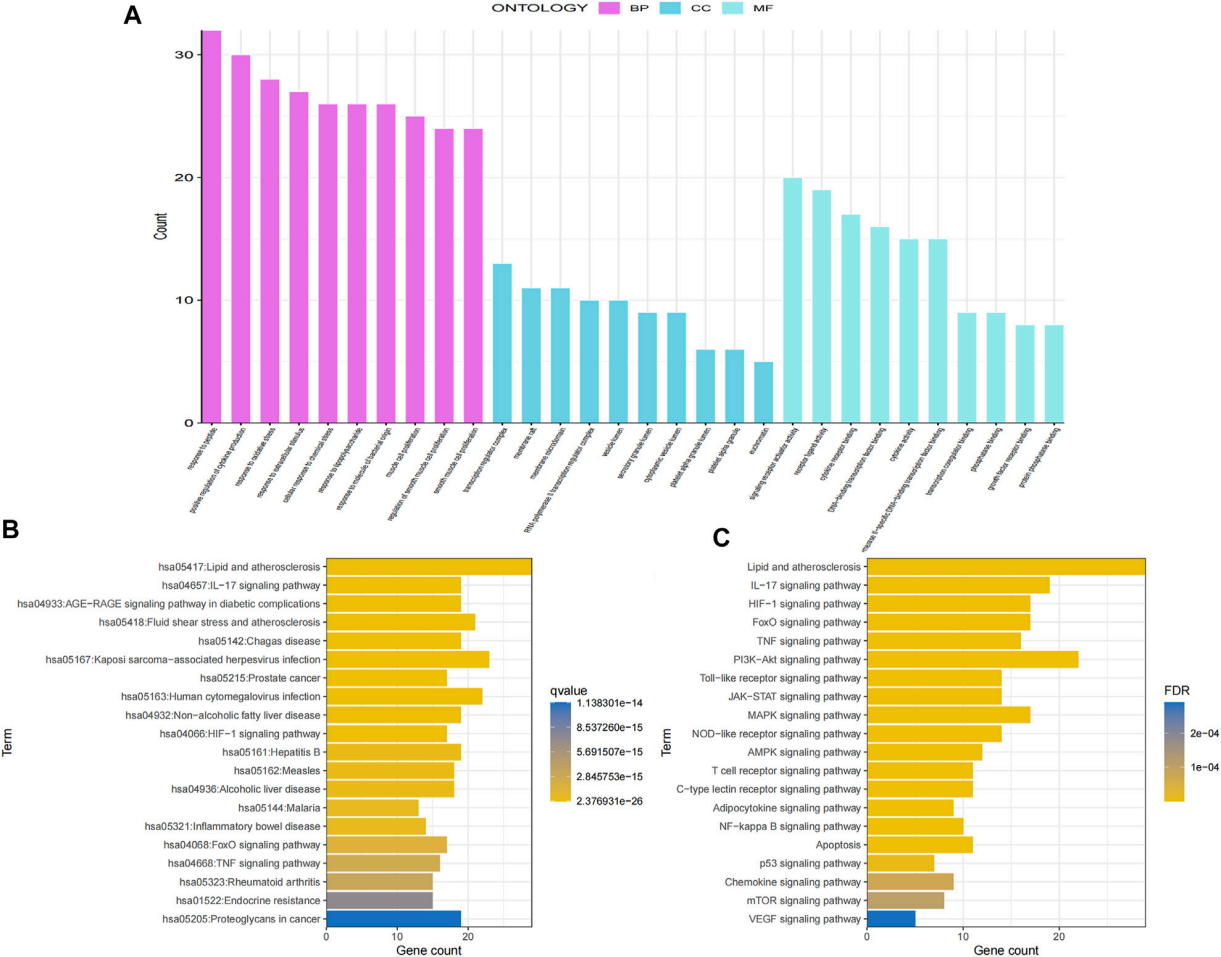
Gene Ontology (GO) and Kyoto Encyclopedia of Genes and Genomes (KEGG) enrichment analyses of potential core targets. **(A)** GO enrichment analysis. **(B)** KEGG enrichment analysis. **(C)** Screening of the top 20 ranked KEGG pathway information.

To explore the crucial biological processes of GSG in the treatment of NAFLD, the GO functional enrichment analyses were performed. The results of GO analysis revealed that the 70 genes were enriched in 2730 GO entries, consisting of 2,594 biological process (BP), 30 cellular components (CC), and 109 molecular functions (MF), and the top 10 entries of BP, CC and MF were shown (Figure 3A). The results showed that GSG targets in NAFLD treatment were mainly enriched in response to biological processes such as lipopolysaccharide (GO:0032496), oxidative stress (GO:0006979), and bacterial-derived factors (GO:0002237). The cellular components were mainly membrane rafts (GO:0045121), membrane microdomains (GO:0098857), and other cellular components. The molecular functions were mainly DNA-binding transcription factor binding (GO:0140297), growth factor receptor binding (GO:0070851), protein phosphatase binding (GO:0019903), and other molecular functions. To investigate the representative signaling pathways associated with the key targets, the KEGG enrichment analysis was performed. And the results showed that 156 significantly enriched signaling pathways were retrieved. The top 20 significantly enriched signaling pathways closely correlated with NAFLD were shown (Figure 3B). However, the results of KEGG enrichment analysis were extensive, mainly including diseases, biological processes, and pathways of action. To further meet the purpose of this experimental study, only the top 20 pathway information was taken to plot the bar chart (Figure 3C). These include signaling pathways such as the NF-κB signaling pathway, TNF signaling pathway, PI3K signaling pathway, and apoptosis, etc.

### 3.6 Passage-target and target-component network diagrams

**FIGURE 4:**
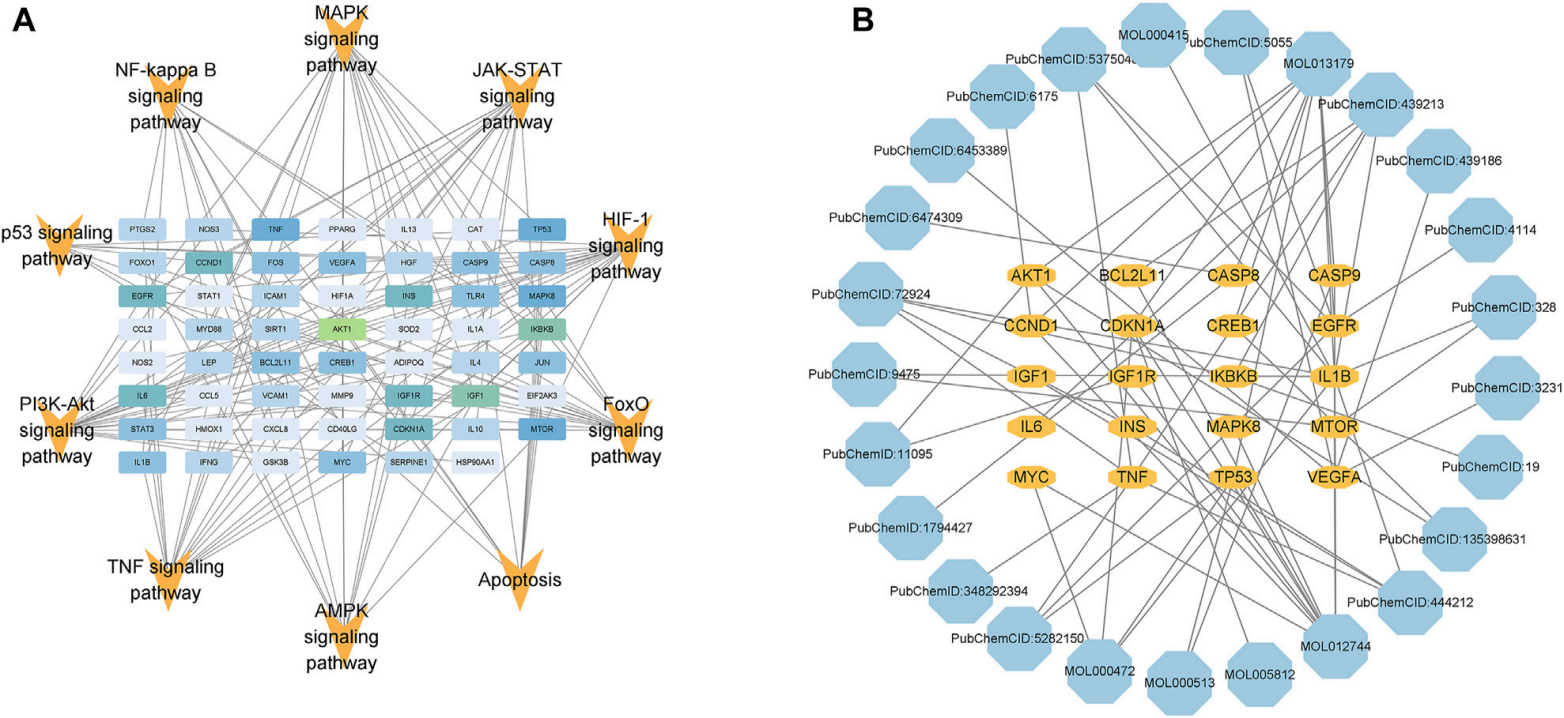
Pathway-target and target-component network diagrams. **(A)** Combined with the pathogenesis of NAFLD, information for 10 pathways and enriched targets was taken for image visualization. **(B)** Based on the degree values of the targets in Figure **(A)**, the top 20 targets and the corresponding component information were taken for image visualization (the color of the compounds changes from light to dark according to the degree values).

In order to screen the core targets and compounds of GSG in the treatment of NAFLD/NASH, 10 signaling pathways closely related to NAFLD/NASH pathogenesis, such as TNF signaling pathway, NF-kappa B signaling pathway, JAK-STAT signaling pathway, and apoptosis, were sorted out (Supplementary Table S4). The targets enriched by these 10 pathways were integrated and imported into Cytoscape 3.9.1 software for visual analysis of the pathway-target network images (Figure 4A). The top 20 targets were selected according to degree values as the core targets of this study and included *AKT1, IKBKB, IL-6, BCL2L11, CASP9, TNF, and CASP8*, etc (Supplementary Table S5). The core targets and their corresponding component information were imported into Cytoscape 3.9.1 software to draw a component-target network diagram (Figure 4B). The top 10 compounds were selected according to degree values as potential core compounds for this study, which included resveratrol, fisetin, D-glucosamine and emodin, etc (Supplementary Table S6).

### 3.7 Molecular docking

**FIGURE 5:**
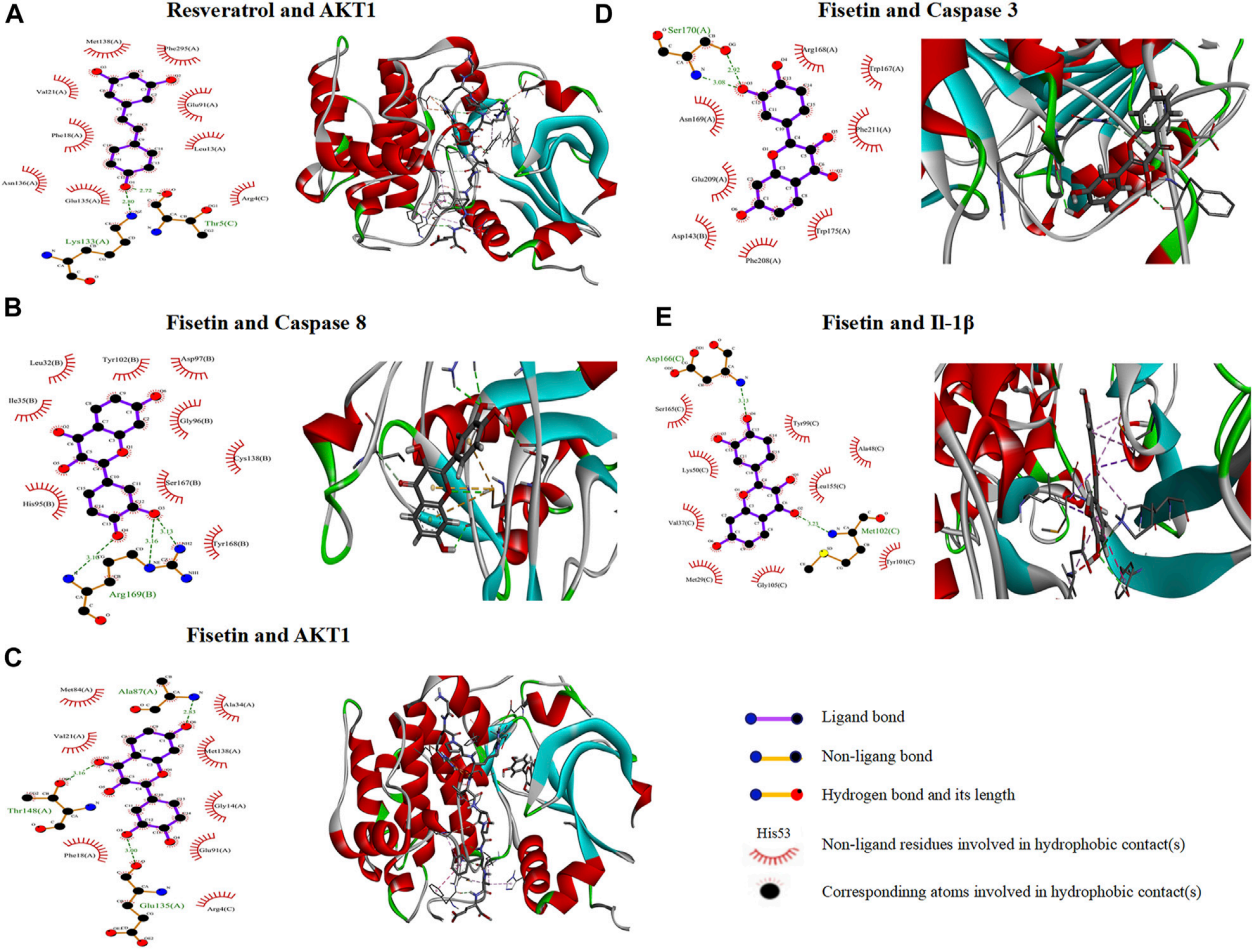
2D and 3D interaction mapping of molecular docking. **(A)** Resveratrol docked with *AKT1*. **(B)** Fisetin docked with *CASP8*. **(C)** Fisetin docked with *AKT1*. **(D)** Fisetin docked with *CASP3*. **(E)** Fisetin docked with *IL-1*β.

Based on the above results, five compounds (resveratrol, fisetin, D-glucosamine, 6-hydroxynicotinic acid, and gallic acid) were screened and molecularly docked with six proteins, that is, CASP8, AKT1, TNF, Bcl2, CASP3, and IL-1β. The docking fractions of the compounds and proteins are shown in the Supplementary Material (Supplementary Table S7). The 3D and 2D images of optimal docking of acceptor and ligand after visualization are shown (Figures 5A–F). The 3D cartoon structure magnifies the ligand–protein binding residue position. 2D visualization of interacting provides the details of docking simulation of key amino acids *via* the H-bonds and hydrophobic interactions. The docking results showed that the two interacted by hydrogen bonding, and resveratrol and fisetin combined well with AKT1, CASP8, CASP3, and IL-1β, respectively. It was shown that a lower affinity represents a higher possibility of binding to both (Liu et al., 2022; Xu et al., 2022). The core ingredients are related to resveratrol and fisetin.

### 3.8 Effect of GSG on liver weight, body weight and the liver index in rats

**FIGURE 6:**
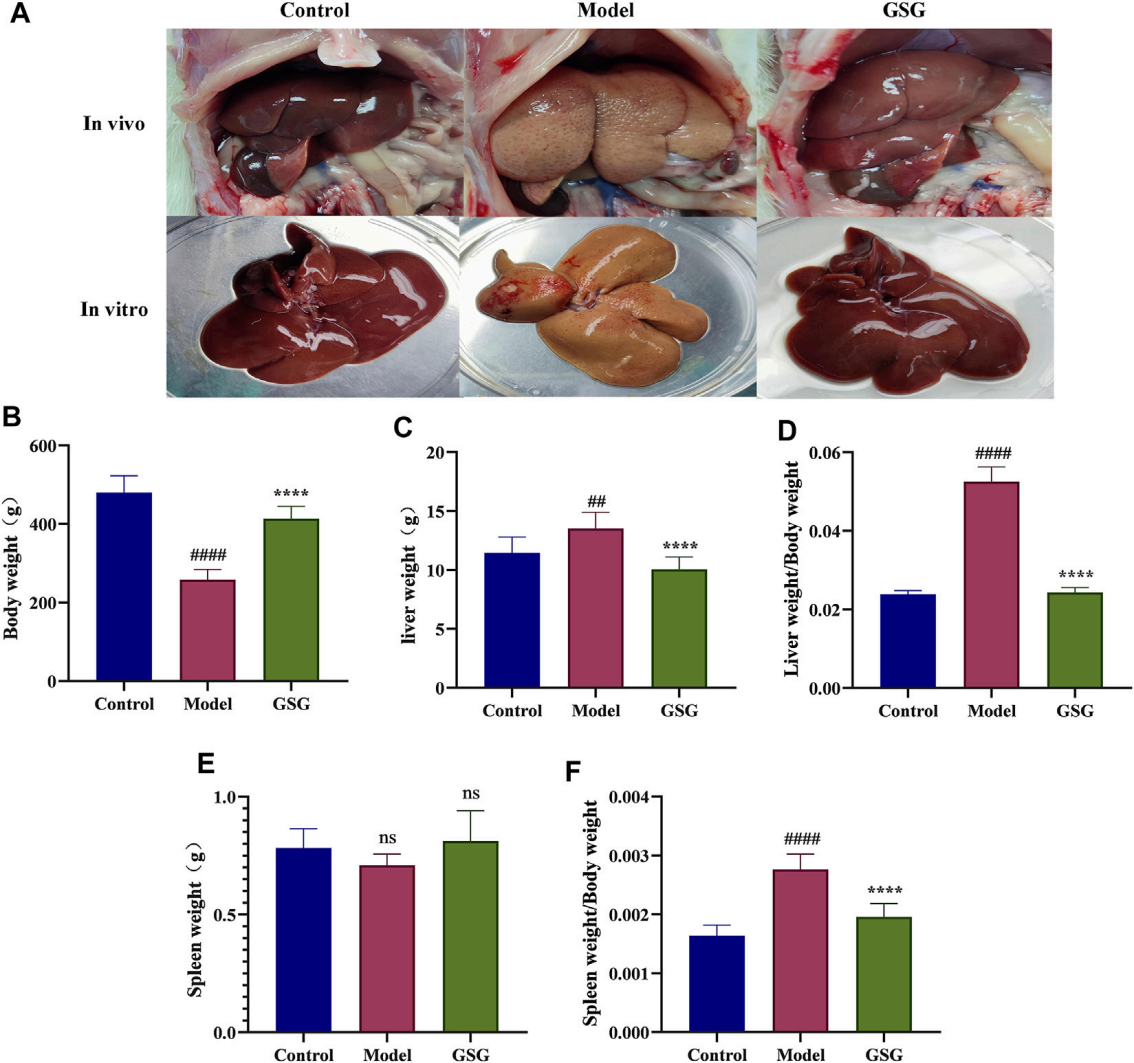
General indicators. **(A)** *in vitro and vivo* plots of the liver in each group. **(B)** Body weight. **(C)** Liver weight. **(D)** Liver index (liver weight/body weight). **(E)** Spleen index (spleen weight/body weight). Data are the mean ± S.E.M.; ^##^ *p* < 0.01, ^####^ *p* < 0.0001 vs. control; ****p < 0.0001 vs. model; *n* = 6.

During the experiment, no death occurred in the control group, while three model rats died and two GSG rats died. The *in vivo* and *vitro* states of the livers of each group of rats were recorded during sampling (Figure 6A). Compared to the control group, the liver of the rats in the model group was lighter in color and the surface of the liver was rougher. The livers of GSG rats showed significant improvement in both color and texture compared with the model group and were more consistent with the direct perception of the livers of control rats. The experimental results showed that the body weight of the model group decreased significantly, and the liver weight, liver index, and spleen index increased significantly compared with those of the control group (Figures 6B–D, F). Compared with the model group, the GSG body weight increased significantly, and the liver weight, liver index, and spleen index decreased significantly (Figures 6B–D, F). The spleen weight of rats in each group showed no significant differences (Figure 6E).

### 3.9 Regulation of abnormal hepatic lipid accumulation by GSG

**FIGURE 7:**
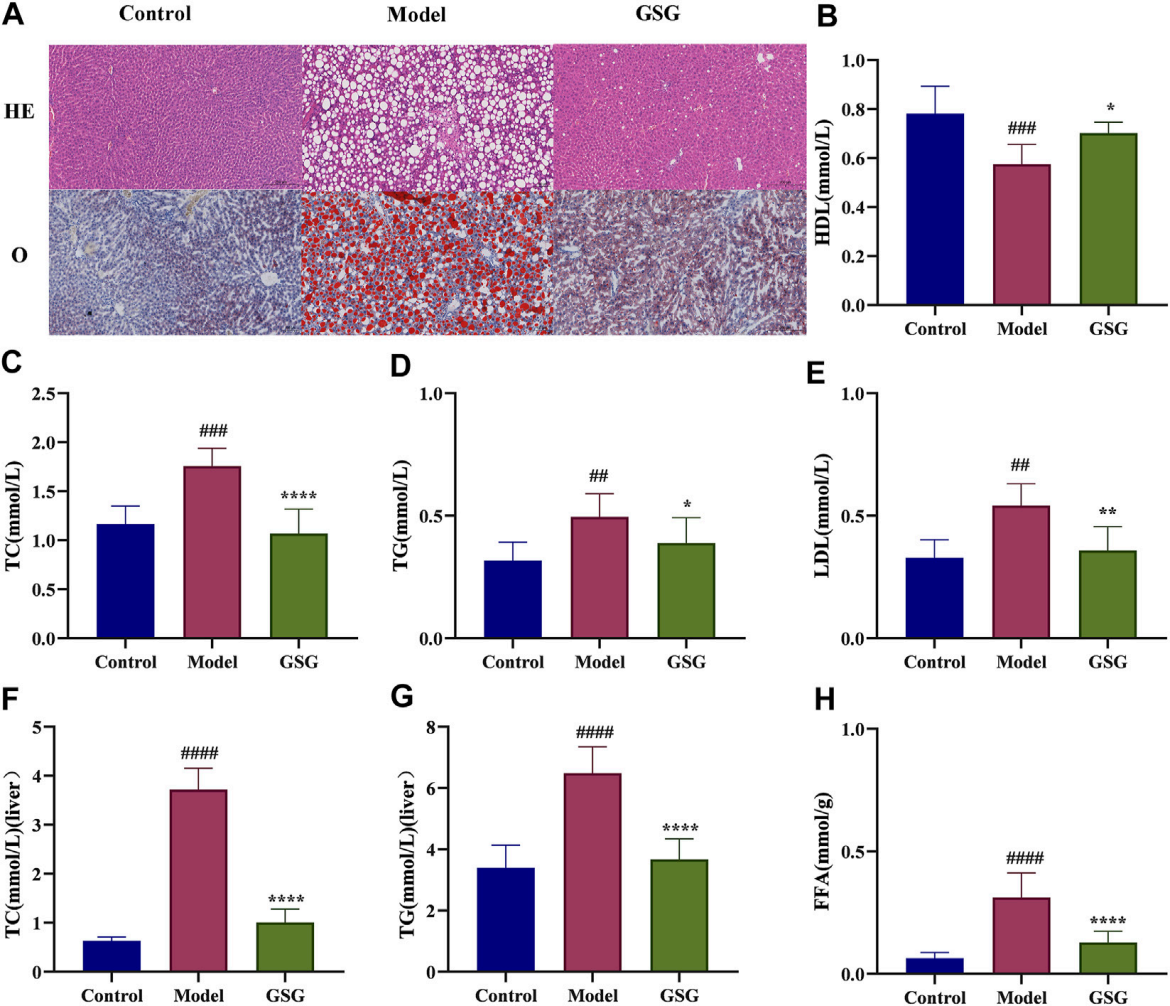
Regulation of lipid metabolism by GSG. **(A)** Oil red O staining in each group. **(B–E)** Serum levels of HDL, TC, TG, and LDL in each group. **(F–H)** Liver tissue levels of TC, TG, and FFA in each group. Data are the mean ± S.E.M.; ^##^ *p* < 0.01, ^####^ *p* < 0.0001 vs. control; *p < 0.05, **p < 0.01, ****p < 0.0001 vs. model; *n* = 6.

It is well known that abnormal lipid metabolism is the basis of NASH formation (Zhang Y. et al., 2015b; Duan et al., 2017), and abnormal hepatic lipid deposition is also an important component of pathological changes in NASH. To investigate the effect of GSG on the lipid metabolism of NASH, histological oil red O staining and the detection of lipid-related indexes in serum and liver were used in this study. The staining results showed that a large number of hepatocytes in the liver of model rats showed bullous steatosis with abnormal lipid accumulation compared with the control group. Lipid accumulation was significantly improved and macrovesicular steatosis of hepatocytes was reduced after GSG drug treatment (Figure 7A). The results of serum and liver tests regarding lipid metabolism indices showed that the levels of TC and TG were significantly higher in the model group than in the control group and were significantly reduced after 8 weeks of GSG treatment (Figures 7C, D, F, G). Moreover, the FFA content in liver tissue (Figure 7H) and the LDL level in serum (Figure 7E) also reflected the same change trend mentioned above, while the changing trend of HDL in serum was the opposite (Figure 7B). The results indicated that GSG alleviated CDHFD-induced hepatocellular steatosis in rats and improved the abnormal accumulation of hepatic lipids.

### 3.10 GSG protects the liver from CDHFD-induced inflammatory evidence, liver injury and liver fibrosis

**FIGURE 8:**
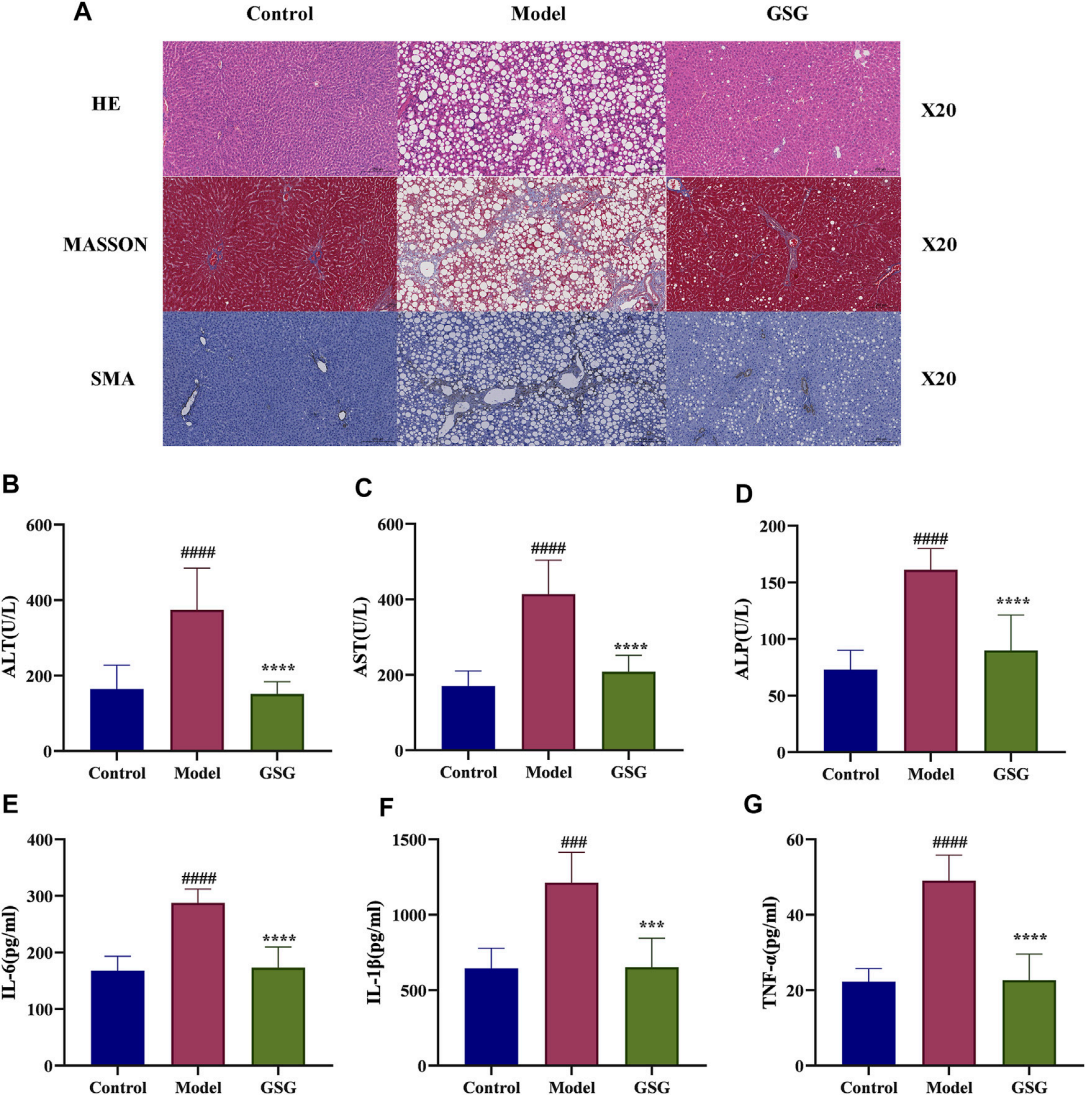
Effects of GSG on liver damage and inflammation. **(A)** Pathological staining and SMA immunohistochemical staining in each group. **(B–D)** Serum levels of ALT, AST, and ALP in each group. **(E–G)** Levels of IL-6, IL-1β, and TNF-α in liver tissue in each group. Data are the mean ± S.E.M.; ^####^ *p* < 0.0001 vs. control; ****p < 0.0001 vs. model; *n* = 6.

To further investigate the effect of GSG on the pathology of NASH model rats, the present study examined the structural changes in the rat liver by HE staining and assessed the degree of liver fibrosis by MASSON staining. The HE staining results showed that compared with the control group, the livers of CDHFD-induced model rats displayed significant hepatocyte swelling, steatosis, and inflammatory cell infiltration. In contrast, GSG treatment improved or even reversed the above pathological changes. In addition, MASSON staining showed that the CDHFD significantly increased the formation of perivascular and interlobular fibrosis in the liver, while GSG attenuated the deposition of collagen fibers in these areas (Figure 8A). HSC activation is a marker of liver fibrosis, while α-SMA is a protein marker of activated HSCs. IHC staining showed that α-SMA expression was significantly increased in the liver tissue of the model rats compared with the control group, while GSG treatment significantly reduced the expression of this protein (Figure 8A). These data suggested that GSG not only improved the pathological liver changes in NASH rats but also effectively inhibited HSC activation and delayed the process of liver fibrosis.

Studies have shown that the elements of disease progression that advance NASH include inflammation, hepatocellular injury, and fibrosis (Barrow et al., 2021). In this experiment, the levels of liver function markers in serum were measured to assess the effect of GSG on liver function injury. The concentrations of ALT, AST, and ALP in the serum of model rats were significantly increased compared with those of the control group. And compared to the model group, the levels of ALT, AST, and ALP in the serum of GSG rats were significantly reduced (Figures 8B–D). Moreover, excessive inflammation has proved to play a crucial role in the progression of NASH (Wang et al., 2020; Barrow et al., 2021). Therefor, the ability to improve this biological process is an important task for new drug development. The levels of IL-6, IL-1β, and TNF-α were significantly increased in the liver of model rats compared with the control group. GSG treatment significantly reduced the levels of inflammatory factors in the liver of the model group (Figures 8E–G). Taken together, the above data suggested that GSG can effectively improve CDHFD-induced liver injury and inflammatory and fibrosis progression in rats.

### 3.11 Effect of GSG on the NF-κB/IκB pathway

**FIGURE 9:**
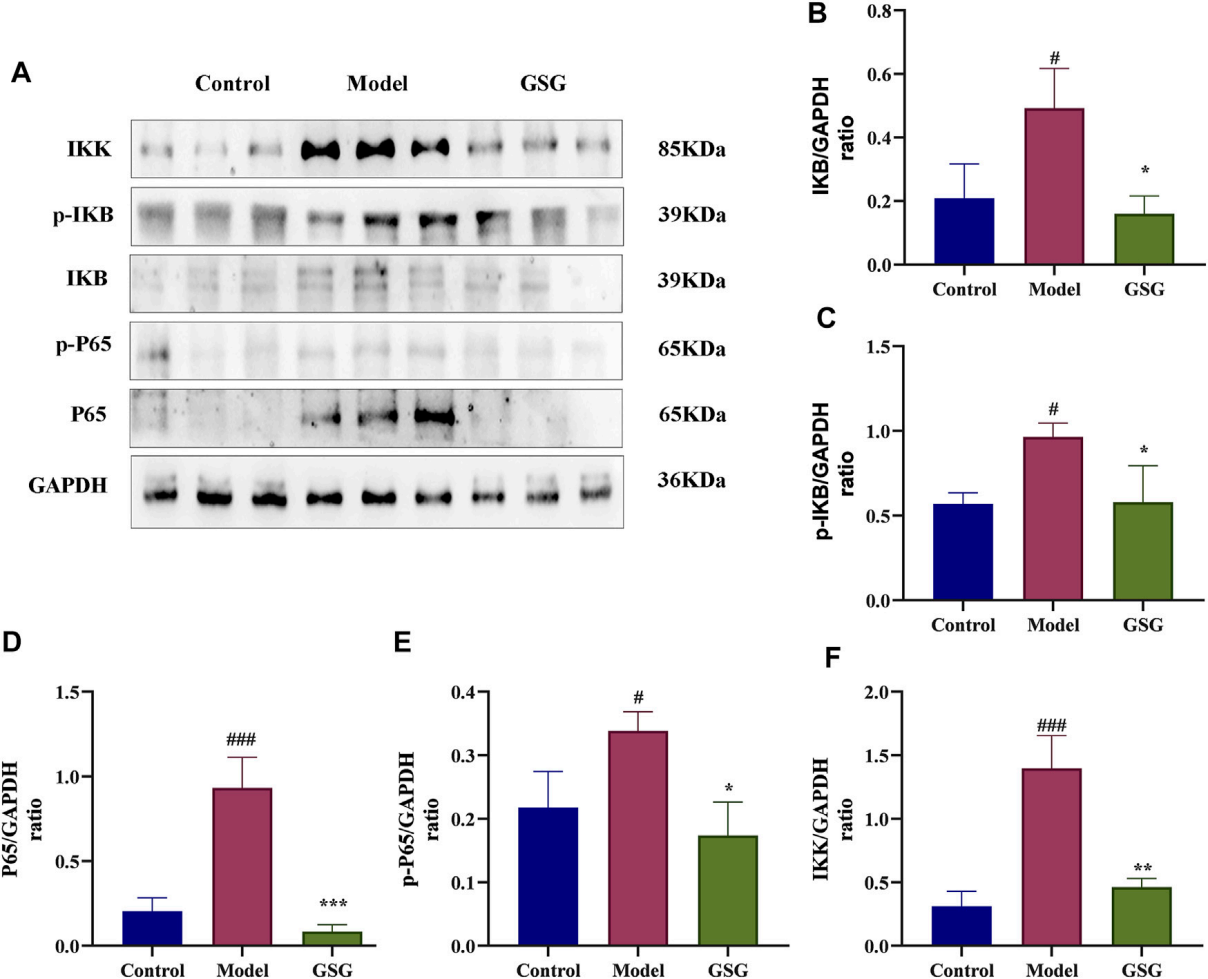
Inhibition of the NF-κB/IκB signaling pathway by GSG in NASH rats. **(A)** Protein blotting bands of P65, p-P65, IκB, p-IκB, and IKK in each group of liver samples. **(B–F)** Grayscale analysis of the protein expression of P65, p-P65, IκB, p-IκB, and IKK in each group of liver samples, using GAPDH as an internal reference. Data are the mean ± S.E.M.; ^#^ *p* < 0.05, ^##^ *p* < 0.01, ^####^ *p* < 0.0001 vs. control; *p < 0.05, **p < 0.01, ***p < 0.001 vs. model; *n* = 6.

It has been shown that the NF-κB pathway is a key signaling pathway for inflammatory reaction and liver injury in steatohepatitis (Xu et al., 2018; Wei et al., 2021). NF-κB is also an important upstream regulator of IL-1β and TNF-α expression (Zhang et al., 2016). Therefore, in order to study the effect of GSG on the NF-κB signaling pathway, the expression of relevant proteins in this pathway was determined by western blotting in this experiment. Compared with control, the expression of protein markers of the NF-κB/IκB signaling pathway, including P65, p-P65, IKB, p-IKB, and IKK, was significantly increased in the liver tissues of NASH rats (Figures 9A–F). The expression of these proteins was significantly decreased after GSG administration. The results indicated that GSG inhibited NF-ĸB signaling pathway activation in the liver tissue of NASH rats.

### 3.12 Effect of GSG on apoptosis

**FIGURE 10:**
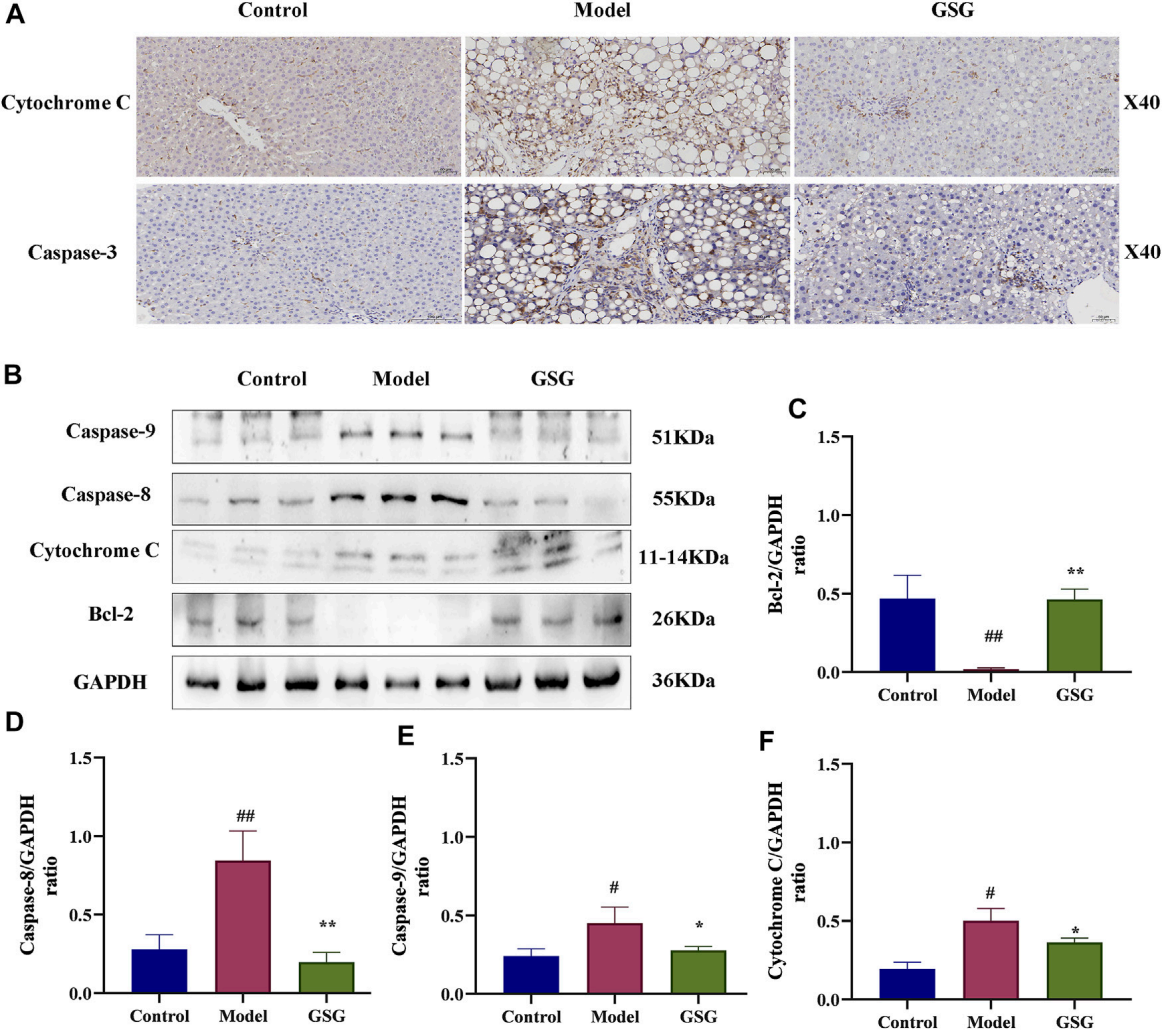
Inhibition of the apoptotic signaling pathway by GSG in NASH rats. **(A)** Protein expression of cytochrome C and caspase-3 in each group of liver samples was investigated by immunohistochemical staining. **(B)** Protein blotting bands of cytochrome C, caspase-8, caspase-9, and Bcl-2 in each group of liver samples. **(C–F)**: Grayscale analysis of the protein expression of cytochrome C, caspase-8, caspase-9, and Bcl-2 in each group of liver samples using GAPDH as an internal reference. Data are the mean ± S.E.M.; ^#^ *p* < 0.05, ^##^ *p* < 0.01 vs. control; *p < 0.05, **p < 0.01 vs. model; *n* = 6.

Apoptosis is a mainly pathological state during NASH (Cohen et al., 2011; Nassir and Ibdah, 2014; Kanda et al., 2018), as well as an important cause of liver injury and a driving factor in the rapid progression of NASH to the cirrhotic stage. Caspases are a conserved family of cysteine proteases that are primarily involved in cell death and inflammation responses. In order to reveal the regulatory effect of GSG on the apoptosis signaling pathway, the expression of apoptosis-related proteins was analyzed by immunohistochemical staining and western blotting. Compared with the control group, the CDHFD significantly activated the protein expression of caspase-3, -8, and -9, and cytochrome C in the liver tissues of NASH rats and inhibited the expression of the anti-apoptotic protein Bcl-2. Compared with the model group, GSG administration reversed the trends of the above mentioned protein expression (Figures 10A–F). Therefore, the present study showed that GSG could effectively inhibit apoptosis and have a protective effect on liver injury.

## 4 Discussion

Owing to a lack of effective drugs, NASH has become a serious health threat worldwide. TCM holds great potential to tackle this health burden. This study suggested that CDHFD successfully induced NASH in rats with massive steatosis, severe inflammatory infiltration, collagen accumulation, and excessive activation of inflammatory pathways. GSG can exert lipid-lowering, anti-inflammatory and delayed liver fibrosis therapeutic effects on NASH by inhibiting the conduction of NF-κB/IκB signaling pathway and their downstream inflammatory and apoptosis signaling.

It is well known that abnormal lipid metabolism is the basis of NAFLD/NASH formation (Zhang Y. et al., 2015b; Duan et al., 2017), and hepatic lipid deposition is an important component of its pathological changes. TC, TG, and FFA were important indicators for assessing the lipid metabolism function of NASH (Manne et al., 2018). NAFLD arises when the uptake of fatty acids (FA) and TG from circulation and *de novo* lipogenesis saturate the rate of FA β-oxidation and very-low density lipoprotein (VLDL)-TG export (Mato et al., 2019). GSG demonstrated its therapeutic effect on improving abnormal lipid accumulation in the liver of NASH by inhibiting the increase of TC, TG, and FFA levels in the liver induced by CDHFD. In fact, the leading cause of death in NASH patients is extrahepatic factors such as cardiovascular disease (Tillman and Rolph, 2020). In patients with NAFLD, insulin resistance causes the liver to produce large amounts of triglyceride-rich VLDL particles, resulting in elevated serum triglycerides. This hyperlipidemia is also accompanied by a decrease in serum HDL levels and an increase in highly atherogenic LDL levels due to elevated VLDL levels. This study found that GSG can reduce the levels of TC, TG, and LDL in the serum of NASH rats, increase the content of HDL, and reflect its regulatory effect on the metabolism of the whole body. These data confirmed the lipid-lowering effect of GSG on both inside and outside the liver.

TCM often shows multiple compounds and multiple targets. Thermo Fisher CD 2.1 was used for data collection from UHPLC-Q/Orbitrap-MS/MS and then the 111 compounds were used for further screening with a mzCloud match score of 80 or higher. GSG may regulate the signaling of NF-κB pathway and apoptotic pathway through active ingredients such as fisetin and resveratrol, with core targets including AKT1, Caspase 8, Caspase 3, and IL-1β, etc. It has been shown that fisetin is a flavonoid polyphenol with antioxidants, anti-inflammatory (Singh et al., 2018); (Huang et al., 2018), and antitumor properties, among other pharmacological effects (Singh et al., 2018). It can not only prevent acetaminophen-induced liver injury by promoting autophagy (Zhang et al., 2020), but also balance the expression of lipid metabolism-related genes by downregulating metabolic disorders and TNF-α/RIPK3 signaling associated with liver inflammation, ultimately achieving the therapeutic effect of inhibiting lipid accumulation and steatohepatitis. Resveratrol is a non-flavonoid phenol found in many plant species (Salehi et al., 2018; Wei and Yu, 2021). *In vitro* and *vivo* studies have shown that resveratrol has biological properties, such as antiaging, antioxidant, cardioprotective, anti-inflammatory and antiplatelet aggregation properties (Izzo et al., 2021). Obesity is a major risk factor for NAFLD, and Poulsen et al. showed that resveratrol reduces diet-induced liver fat accumulation by increasing fatty acid oxidation and reducing lipogenesis (Poulsen et al., 2012). In addition, resveratrol can also alleviate NAFLD-induced liver damage by inhibiting autophagy and signaling of the IκBα-NF-κB pathway (Li et al., 2014) and downregulating the protein levels of IL-6, IL-1β, and TNF-α (Che et al., 2020). Therefore, the active ingredients of GSG have the functions of lowering lipids, anti-inflammatory, and regulating inflammatory pathways.

The NF-κB pathway has been reported to be a key player not only in the progression but also in the initiation of NAFLD. Under normal physiological conditions, NF-κB forms complexes with its inhibitors IκBs (α or β) and is maintained in this inactive state in the cytoplasmic matrix. When IκB is phosphorylated by IKKα or IKKβ, NF-κB is released and translocates to the nucleus, followed by the release of large amounts of proinflammatory cytokines (Luo et al., 2015). Thus, TNF-α and IL-1β promote NF-κB activation (Diehl et al., 2005; Chen et al., 2020), and NF-κB is also an important upstream regulatory protein that enhances TNF-α and IL-1β production (Zhang et al., 2016). This continuous cycle of the inflammatory response ultimately leads to structural damage and liver dysfunction (Yang and Seki, 2015; Zheng et al., 2019). At the same time, the repeated extension of inflammation leads to an increase in the accumulation of extracellular matrix, thereby exacerbating the progression of liver inflammation and liver fibrosis. The results showed that GSG significantly reduced the levels of TNF-α, IL-6, and IL-1β in the liver, improved the expression of fibrosis α-SMA in the liver, and inhibited the protein expression of P65, p-P65, IKB, p-IKB, and IKK in liver tissues. Therefore, the NF-κB/IκB signaling pathway may be the core mechanism of GSG-mediated anti-inflammatory and retarding liver fibrosis efficacy.

Apoptosis is a highly organized and genetically controlled form of cell death (Alkhouri et al., 2011). It is not only a major form of hepatocyte injury during the course of NASH but also a key mechanism for accelerating inflammation and fibrosis in NASH (Hatting et al., 2013). Bcl-2 and Bax are the core genes that initiate the mitochondrial pathway for endogenous apoptosis, and there are specific binding sites for NF-κB on the promoter of Bcl-2. Consistently, recent evidence indicated that NF-κB can induce apoptosis by directly downregulating Bcl-2 expression through the transcriptional pathway (Xie et al., 2017; Quan et al., 2019). It has been evidenced that the intrinsic apoptotic pathway could be triggered by cellular stress signals (e.g., DNA damage and cytokines) (Bao and Shi, 2007). The opening of the pathway is marked by the release of cytochrome c into the cytoplasmic matrix, which then constitutes the apoptosome through its interaction with apoptosis protease activating factor 1 (Apaf-1) and procaspase-9 (Riedl and Salvesen, 2007). The assembly of the apoptosome then leads to caspase-9 activation, which further activates caspase-3 for apoptosis execution (Galluzzi et al., 2018). Caspase-8 can directly activate caspase-3 in response to receptor-mediated apoptotic stimuli or enhance this apoptotic signaling with the help of endogenous apoptotic pathways (Mandal et al., 2014). ALT, AST, and ALP are important indicators for assessing liver damage. Studies have shown that GSG can significantly reduce the content of ALT, AST, and ALP in the serum of NASH rats and has the effect of improving liver damage. In addition, GSG also significantly inhibited the protein expression of Caspase-3, 8, 9, and Cytochrome C in NASH rat liver tissue and increased the protein expression of Bcl-2. Actually, GSG significantly alleviated the rat NASH, mechanistically by regulating the NF-κB/IκB signaling pathway and its downstream inflammatory and apoptosis signaling.

However, the current study still has some limitations. For example, this experiment lacked research data on different concentrations of GSG and the comparison of efficacy results with commonly used drugs for NASH. Moreover, the therapeutic effect of the core ingredients proposed in this study on NASH also needs to be further verified *in vitro* and *vivo*.

## 5 Conclusion

In conclusion, the experimental results show that the molecular mechanism of GSG in the treatment of NASH is mainly reflected in the inhibition of the NF-κB signaling pathway and its downstream inflammatory and apoptosis signals. This research experience of GSG for NASH not only reflects the medicinal effects of GSG in lipid lowering, anti-inflammation, and inhibition of hepatocyte apoptosis but also provides data support to confirm the therapeutic advantages of Chinese medicine with multicomponent, multitarget, and multi-pathway effects. This multidisciplinary research method also provides new ideas for modern pharmacological research of Chinese medicine.

## Data Availability

The original contributions presented in the study are included in the article/Supplementary Materials, further inquiries can be directed to the corresponding author.
