# Supplementary material for: Exploring the molecular mechanism of Gan Shuang granules for the treatment of non-alcoholic steatohepatitis using network pharmacology, molecular docking, and experimental verification
Source: Front Pharmacol. 2023 Jan 24;14:1082451. doi: 10.3389/fphar.2023.1082451 (PMC9902723; doi:10.3389/fphar.2023.1082451)
Supplement: Supplementary file 1 [file DataSheet1.docx]

Supplementary Material

Exploring the molecular mechanism of GanShuang granules for the treatment of nonalcoholic steatohepatitis using network pharmacology, molecular docking, and experimental verification

Guoguo Zhi^1^, †, Bingjie Shao^1^, †, Tianyan Zheng^1^, Jie Mu^1^, Jingwei Li^1^, Yiyuan Feng^1^, Sha Zhu^1^, Yanni Dang^2^, Feng Liu^2*^, Dong Wang^1*^

*** Corresponding Author:**

Wang Dong, Email: [wangdong@cdutcm.edu.cn](mailto:wangdong@cdutcm.edu.cn)

Liu Feng, Email: liufeng1720@163.com

† These authors contributed equally to this work and share first authorship

# Supplementary Figures


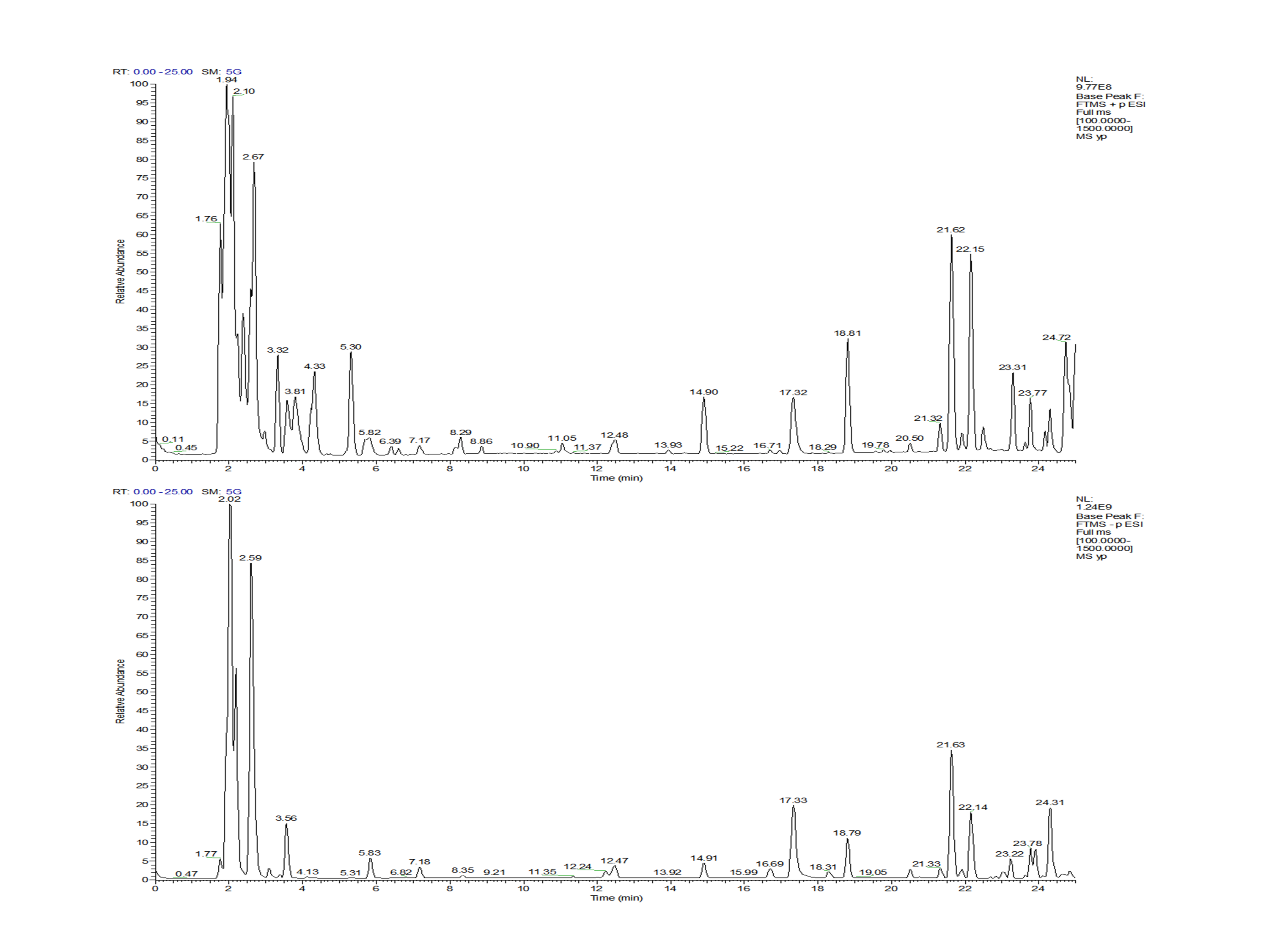


**Supplementary Figure 1.** Total ion current chromatograms (TICCs) for UHPLC-Q/Orbitrap-MS/MS analysis


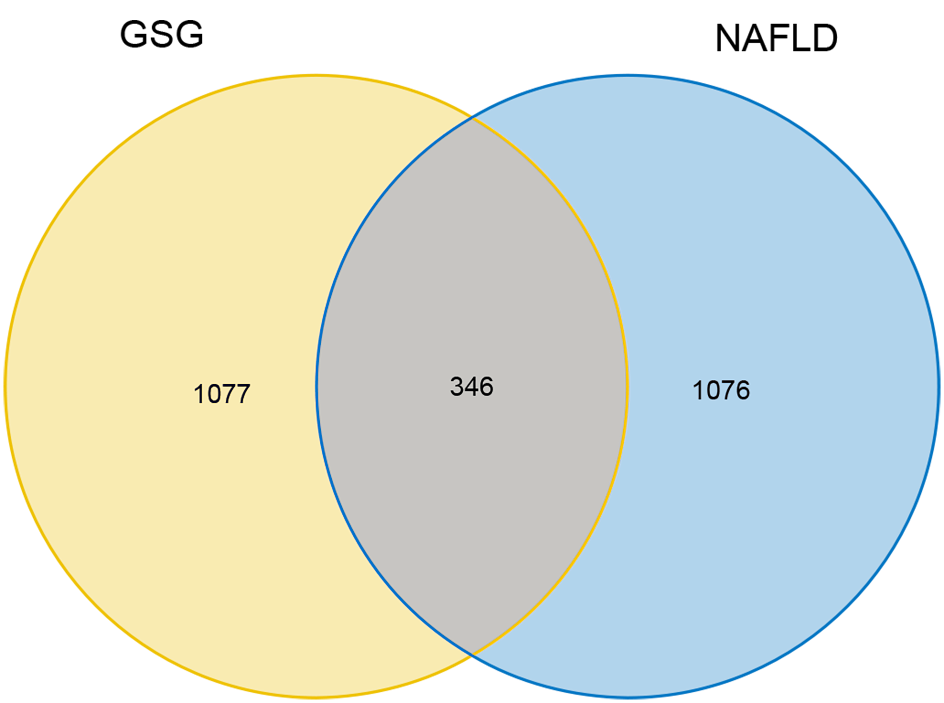


**Supplementary Figure 2.** The intersection of GanShuang Granules (GSG) and non-alcoholic fatty liver disease (NAFLD) targets

# Supplementary Tables

**Supplementary Tables 1.** Analysis of the top 10 components of mzCloud Best Match scores based on UHPLC-Q/Orbitrap-MS/MS

| Name | Formula | Molecular Weight | RT [min] | mzCloud  Best Match |
| --- | --- | --- | --- | --- |
| Nicotinic acid | C6 H5 N O2 | 123.03217 | 2.462 | 95.7 |
| Resveratrol | C14 H12 O3 | 228.07843 | 18.806 | 94.1 |
| Senkyunolide H | C12 H16 O4 | 224.10479 | 23.001 | 93.6 |
| Adenosine | C10 H13 N5 O4 | 267.09648 | 3.317 | 93.4 |
| cis-Resveratrol | C14 H12 O3 | 228.07848 | 16.753 | 92.7 |
| Methyl caffeate | C10 H10 O4 | 194.05752 | 22.15 | 92.5 |
| DL-Stachydrine | C7 H13 N O2 | 143.09449 | 1.994 | 92.5 |
| Chlorogenic acid | C16 H18 O9 | 354.09499 | 12.733 | 92.5 |
| Trigonelline | C7 H7 N O2 | 137.04747 | 1.936 | 92.2 |
| Guanine | C5 H5 N5 O | 151.0493 | 2.175 | 92.2 |

**Supplementary Tables 2.** Take the top 10 ingredient information according to degree

| ID | Name | Degree | |
| --- | --- | --- | --- |
| PubChemCID:9475 | DL-Norleucine | | 83 |
| PubChemCID:328 | 4-Hydroxymandelicacid | | 77 |
| MOL012744 | Resveratrol | | 67 |
| PubChemCID:10691 | Azelaicacid | | 63 |
| PubChemCID:444212 | trans-Aconiticacid | | 52 |
| PubChemCID:72924 | 6-Hydroxynicotinicacid | | 35 |
| PubChemCID:439213 | D-Glucosamine | | 32 |
| PubChemCID:5375048 | Indole-3-acrylicacid | | 29 |
| PubChemID:11095 | 2-(3,4-dihydroxyphenyl)-5,7-dihydroxy-3,4-dihydro-2H-1-benzopyran-4-one | | 26 |
| MOL013179 | Fisetin | | 24 |

**Supplementary Tables 3.** MCODE scores for PPI subnetworks

| Cluster | Score | Nodes | Edges |
| --- | --- | --- | --- |
| 1 | 53.623 | 70 | 1850 |
| 2 | 7.474 | 39 | 142 |
| 3 | 5.875 | 17 | 47 |
| 4 | 5.368 | 20 | 51 |
| 5 | 4.8 | 6 | 12 |
| 6 | 4.5 | 5 | 9 |
| 7 | 4.5 | 5 | 9 |
| 8 | 3.333 | 4 | 5 |
| 9 | 3.333 | 4 | 5 |
| 10 | 3.143 | 8 | 11 |
| 11 | 3 | 5 | 6 |
| 12 | 3 | 3 | 3 |
| 13 | 3 | 3 | 3 |
| 14 | 2.5 | 5 | 5 |

**Supplementary Tables 4.** Combined with KEGG enrichment analysis and literature research, the top 10 pathway information was obtained

| ID | Description | GeneRatio | qvalue |
| --- | --- | --- | --- |
| hsa04668 | TNF signaling pathway | 16/70 | 2.66E-15 |
| hsa04064 | NF-kappa B signaling pathway | 10/70 | 1.61E-08 |
| hsa04630 | JAK-STAT signaling pathway | 14/70 | 9.68E-11 |
| hsa04210 | Apoptosis | 11/70 | 1.77E-08 |
| hsa04151 | PI3K-Akt signaling pathway | 22/70 | 1.79E-13 |
| hsa04068 | FoxO signaling pathway | 17/70 | 1.66E-15 |
| hsa04010 | MAPK signaling pathway | 17/70 | 3.82E-10 |
| hsa04152 | AMPK signaling pathway | 12/70 | 4.87E-10 |
| hsa04115 | p53 signaling pathway | 7/70 | 1.95E-06 |
| hsa04151 | PI3K-Akt signaling pathway | 22/70 | 1.79E-13 |

**Supplementary Tables 5.** Core targets screened by degree

| Gene | Degree | Gene | Degree |
| --- | --- | --- | --- |
| AKT1 | 8 | MTOR | 4 |
| IGF1 | 6 | TNF | 4 |
| IKBKB | 6 | TP53 | 4 |
| CCND1 | 5 | BCL2L11 | 3 |
| CDKN1A | 5 | CASP8 | 3 |
| EGFR | 5 | CASP9 | 3 |
| IGF1R | 5 | CREB1 | 3 |
| IL6 | 5 | IL1B | 3 |
| INS | 5 | MYC | 3 |
| MAPK8 | 4 | VEGFA | 3 |

**Supplementary Tables 6.** Core components screened by degree

| Node_ID | Name | Degree |
| --- | --- | --- |
| MOL012744 | Resveratrol | 12 |
| MOL013179 | Fisetin | 9 |
| PubChemCID:439213 | D-Glucosamine | 5 |
| PubChemCID:444212 | trans-Aconiticacid | 4 |
| PubChemCID:72924 | 6-Hydroxynicotinicacid | 4 |
| MOL000472 | Emodin | 4 |
| PubChemCID:5282150 | Rhoifolin | 3 |
| PubChemCID:5375048 | Indole-3-acrylicacid | 3 |
| PubChemCID:5055 | Resorcinolmonoacetate | 2 |
| PubChemID:11095 | 2-(3,4-dihydroxyphenyl)-5,7-dihydroxy-3,4-dihydro-2H-1-benzopyran-4-one | 2 |

**Supplementary Tables 7.** The binding force between the receptor and the ligand

|  | Resveratrol | Fisetin | D-Glucosamine | 6-Hydroxynicotinicacid | Gallic acid |
| --- | --- | --- | --- | --- | --- |
| Caspase 8 | -5.8 | -7 | -4.7 | -4.6 | -5.4 |
| AKT1 | -7.8 | -9.4 | -5.6 | -6.1 | -6.4 |
| TNF | -6.2 | -6 | -4.9 | -5.6 | -6 |
| Bcl2 | -6.1 | -6.3 | -4.2 | -4.3 | -4.5 |
| Caspase 3 | -6.3 | -6.9 | -5.2 | -4.8 | -5.3 |
| IL1β | -6.5 | -6.8 | -5.1 | -5.4 | -5.9 |
